# Resection of a solitary bone metastasis using preoperative computed tomography (CT)-guided hook wire localization: a case report

**DOI:** 10.11604/pamj.2022.42.264.35146

**Published:** 2022-08-10

**Authors:** Benjamin Augereau, Nicolas Pointet, Pier-Olivier Duboe, Tanguy Vendeuvre

**Affiliations:** 1 Department of Orthopaedic Surgery, University Hospital, Poitiers, France,; 2 Department of Radiology, University Hospital, Poitiers, France,; 3 Department of Orthopaedic Surgery, Spine-Neurostimulation Unit, *Institut P’ Centre National de la Recherche Scientifique, Unité Propre de Recherche (CNRS UPR)*, University Hospital, Poitiers, France

**Keywords:** Bone metastasis, hook-wire, computed tomography, case report

## Abstract

Secondary bone metastasis are a common evolution of many types of cancer. In some instance, it is needed to remove those metastases to improve the prognosis of the patient. Bone metastasis that are invisible and non-palpable during the intervention are difficult to remove while being sure to respect safe resection margins. We present the case of a woman suffering from breast cancer. Despite the treatment of the primitive lesion, she presented a solitary bone metastasis on her iliac crest. The lesion was removed surgically but could not be seen or touched during the procedure due to its location. To be certain to remove this lesion during surgery, while respecting safe resection margins, we performed a pre-operative computed tomography (CT)-guided hook wire localization. Using this method, the anatomopathological examination confirmed the tumoral nature of the lesion and that the resection margins were in a safe zone. This technique frequently used to target soft lesion in cases of breast or lung cancers could therefore be used the same way with solid bone lesion.

## Introduction

Bone metastases are frequently associated with all advanced solid cancers and particularly in case of prostate, breast, and lung cancers [1]. In cases of limited bone spreading, a curative treatment of these metastasis could improve the long-term prognosis of patients [2,3]. To remove the lesion, two options are available: percutaneous ablation using radiofrequency, cryoablation or microwaves, or surgical resection. While the first option is suited for pain relief, in most case it doesn´t remove the cancerous cells completely and doesn´t stop the cancer from spreading furthermore [4,5]. To prevent recurrence or spreading of the cancer, it is required to perform a complete resection of the lesion including safe margins [2]. Percutaneous ablation is commonly performed under X-ray or computed tomography guidance. But finding the exact location of the tumor during a surgical procedure can be difficult if it´s not visible or palpable. Previous reports have already described a hook guided technique to target lesion in cases of breast or lung tumors [6,7]. We propose here a method to locate and remove a solitary bone metastasis, based on the pre-operative placement of a hook on either side of the metastasis.

## Patient and observation

**Patient information:** this case concerns a 51-year-old female patient presenting with breast cancer. The primitive lesion was operated. It corresponded to a 42 mm ductal carcinoma. The work-up revealed mobile axillary adenopathy and distant bone metastases. The tumor analysis showed hormone receptor positivity. The tumor was therefore classified as T2N1M1 (Stage 4) RE+, RP+. The patient received additional treatment by radiotherapy, chemotherapy, and hormonal treatment.

**Figure 1:**
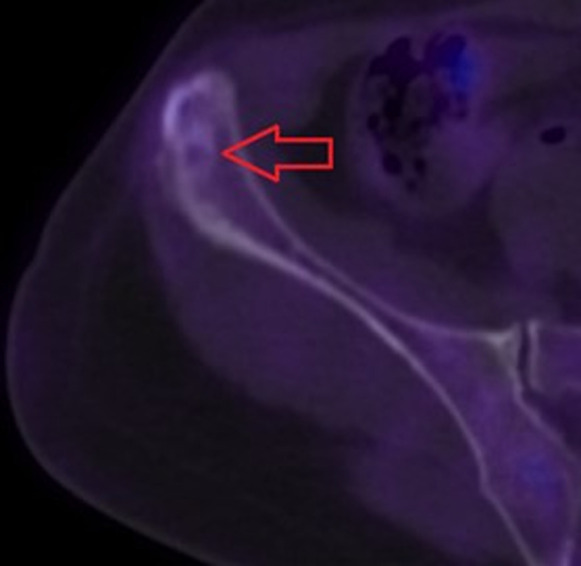
persistent bone metastases on the right pelvic bone detected using bone PET-CT

**Clinical findings:** after initial and adjuvant treatment, the extension of the disease was evaluated by a whole-body CT and a Positron emission tomography (PET)-CT. A unique secondary bone metastasis on the right iliac crest was discovered (Figure 1).

**Diagnostic assessment:** a multidisciplinary staff was held to decide the best course of action concerning this solitary lesion. Because of its relative proximity with the skin, radiologists did not consider thermo-ablation to be the best option due to the risk of skin burns. It was decided to carry out a surgical resection of this secondary lesion to assure the best outcome possible. Due to its location, the lesion was not palpable nor visible during the surgery. We would, therefore, be taking the risk of not removing it completely. It was proposed to perform a preoperative location of the lesion using hook wire placement under CT guidance.

**Figure 2:**
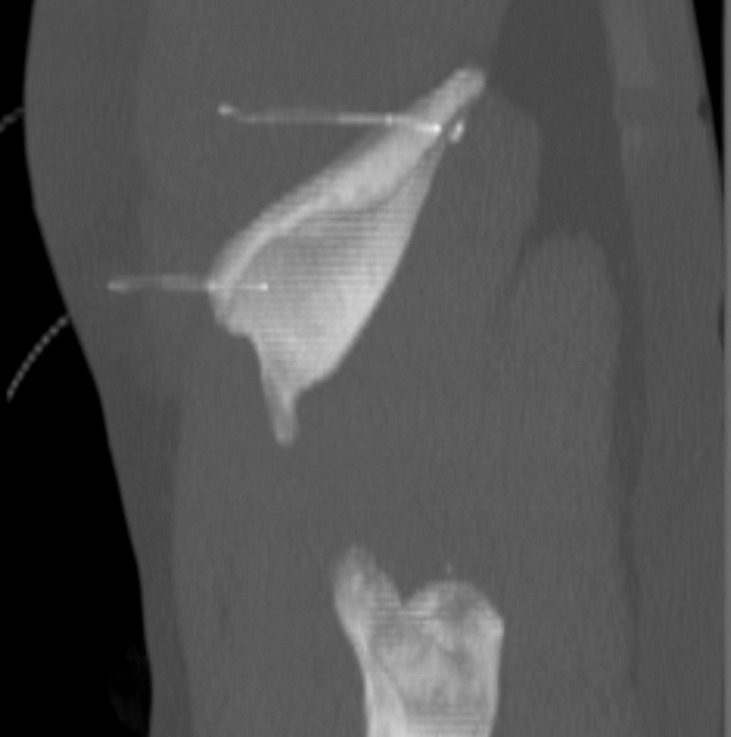
pre-operative targeting of the metastasis using hook wires placed, under CT-guidance, on both sides of the lesion

**Figure 3:**
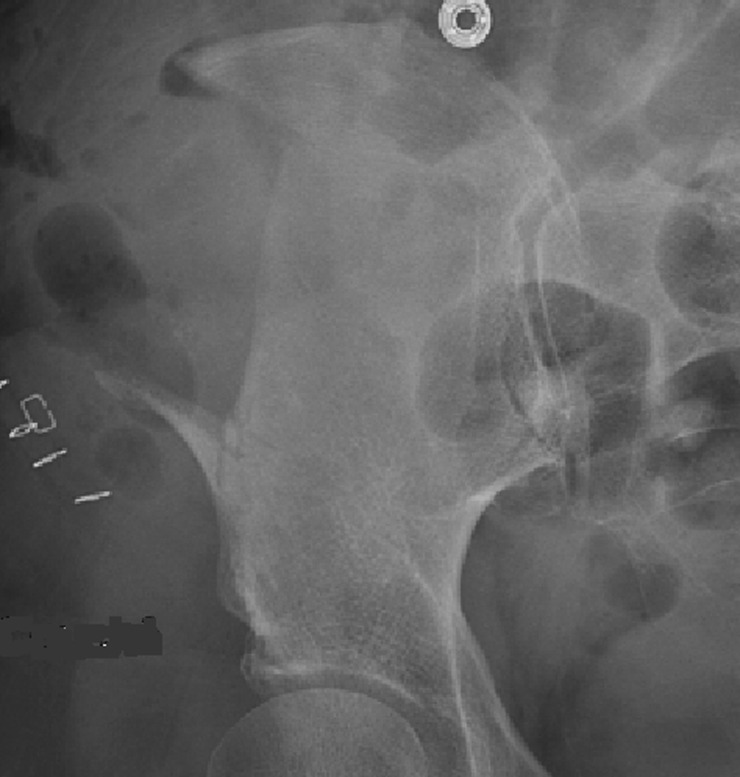
postoperative X-ray showing the partial pelvectomy and complete removal of the lesion

**Therapeutic intervention:** after local anesthesia, under CT guidance, we first targeted the bone two cm below the lower edge of the metastasis and drilled it using a Bonopty biopsy needle (Bonopty®, AprloMed AB, Uppsala, Sweden). A hook wire (Cook, Inc. Bjaeverskov, Denmark) was then inserted into the needle and placed in the soft tissues behind the iliac bone. We repeated the same procedure to place another hook 2 cm above the top edge of the metastasis (Figure 2). A complete surgical resection of the lesion was performed following the hook guidance (Figure 3). The surgery lasted 20 minutes and the blood loss was approximately 50cc.

**Follow up and outcomes:** pathology examination confirmed that the metastases originated from the primitive breast tumor. Surgical margins of 2 cm were observed according to the presurgical planning. No complications were noted in the post-operative period, the wound was clean and showed no signs of infection. At the last follow up positron emission tomography (PET)-CT, one year after the surgery, there was no signs of recurrence, and the patient expressed no pain at the site of the surgery. She did not suffer any loss of function in connection with the surgery and was satisfied with the outcome of the procedure.

## Discussion

Bone metastases are common in advanced solid cancers. Surgical resection is sometimes difficult, for instance in cases of small or non-palpable lesions. This case report showed the effectiveness of a non-palpable bone metastasis targeting using CT-guided hook wires placement. We did not find a research paper or communication about this subject. Yet it is a simple and reproducible procedure that could help the management of small bone lesions that could not be seen or touched during the surgery. This case report also underlines the crucial importance of a multidisciplinary discussion of the cases to generate innovative solutions.

## Conclusion

Pre-operative CT-guided hook wire placement is a technique that ensures that deep bone metastases can be removed in a reliable and reproductible way with the least possible invasiveness. Therefore, this procedure would be a good addition to the therapeutic arsenal already available in the management of primary or secondary bone cancer.

## References

[1] Debiais F (2015). Données épidémiologiques et cliniques des métastases osseuses. Oncologie.

[2] Ratasvuori M, Wedin R, Hansen BH, Keller J, Trovik C, Zaikova O (2014). Prognostic role of en-bloc resection and late onset of bone metastasis in patients with bone-seeking carcinomas of the kidney, breast, lung, and prostate: SSG study on 672 operated skeletal metastases. J Surg Oncol.

[3] Dürr HR, Müller PE, Lenz T, Baur A, Jansson V, Refior HJ (2002). Surgical treatment of bone metastases in patients with breast cancer. Clin Orthop Relat Res.

[4] Link TM, de Mayo R, O´Donnell RJ (2007). Radiofrequency ablation-an alternative for definitive treatment of solitary bone metastases. Eur Radiol.

[5] Santiago FR, del Mar Castellano García M, Montes JLM, García MR, Fernández JMT (2009). Treatment of bone tumours by radiofrequency thermal ablation. Curr Rev Musculoskelet Med.

[6] Dimitrovska MJ, Mitreska N, Lazareska M, Jovanovska ES, Dodevski A, Stojkoski A (2015). Hook wire localization procedure and early detection of breast cancer-our experience. Open Access Maced J Med Sci.

[7] Jortay AM, Daled H, Faverly D (1999). Contribution of hook-guided breast biopsy to the pathological diagnosis of mammographic lesions. Acta Chir Belg.

